# JWH-182: a safe and effective synthetic cannabinoid for chemotherapy-induced neuropathic pain in preclinical models

**DOI:** 10.1038/s41598-024-67154-y

**Published:** 2024-07-15

**Authors:** Leontina-Elena Filipiuc, Ioana Creangă-Murariu, Bogdan-Ionel Tamba, Daniela-Carmen Ababei, Răzvan-Nicolae Rusu, Gabriela-Dumitrița Stanciu, Raluca Ștefanescu, Mitică Ciorpac, Andrei Szilagyi, Raluca Gogu, Silviu-Iulian Filipiuc, Ivona-Maria Tudorancea, Carmen Solcan, Teodora Alexa-Stratulat, Marinela-Carmen Cumpăt, Doina-Clementina Cojocaru, Veronica Bild

**Affiliations:** 1 Advanced Research and Development Center for Experimental Medicine (CEMEX), “Grigore T. Popa” University of Medicine and Pharmacy, University Street No. 16, 700115 Iasi, Romania; 2 Department of Pharmacology, Clinical Pharmacology and Algesiology, “Grigore T. Popa” University of Medicine and Pharmacy, University Street No. 16, 700115 Iasi, Romania; 3 Pharmacodynamics and Clinical Pharmacy Department, “Grigore T. Popa” University of Medicine and Pharmacy, University Street No. 16, 700115 Iasi, Romania; 4 Faculty of Veterinary Medicine, “Ion Ionescu de La Brad” University of Life Sciences, 700490 Iasi, Romania; 5 Department of Medical Specialties I and III, “Grigore T. Popa” University of Medicine and Pharmacy, University Street No. 16, 700115 Iasi, Romania; 6 Clinical Rehabilitation Hospital, Cardiovascular and Respiratory Rehabilitation Clinic, Pantelimon Halipa Street No. 14, 700661 Iasi, Romania; 7 Center of Biomedical Research, Romanian Academy, Iasi Branch, Iasi, Romania; 8 Oncology Department, Regional Institute of Oncology, Iasi, Romania; 9 Department of Medical Oncology-Radiotherapy, “Grigore T. Popa” University of Medicine and Pharmacy, University Street No. 16, 700115 Iasi, Romania

**Keywords:** Synthetic cannabinoids, In-vivo acute toxicity, JWH-182, In-vitro toxicity, Neuropathic pain, Paclitaxel-induced neuropathic pain, Drug development, Experimental models of disease, Preclinical research

## Abstract

Chemotherapy-induced neuropathic pain (CINP), a condition with unmet treatment needs, affects over half of cancer patients treated with chemotherapeutics. Researchers have recently focused on the endocannabinoid system because of its critical role in regulating our bodies' most important functions, including pain. We used in vitro and in vivo methods to determine the toxicity profile of a synthetic cannabinoid, JWH-182, and whether it could be potentially effective for CINP alleviation. In vitro, we evaluated JWH-182 general toxicity, measuring fibroblast viability treated with various concentrations of compound, and its neuroprotection on dorsal root ganglion neurons treated with paclitaxel. In vivo, we performed an evaluation of acute and 28-day repeated dose toxicity in mice, with monitoring of health status and a complete histopathological examination. Finally, we evaluated the efficacy of JWH-182 on a CINP model in mice using specific pain assessment tests. JWH-182 has an acceptable toxicity profile, in both, in vitro and in vivo studies and it was able to significantly reduce pain perception in a CINP model in mice. However, the translation of these results to the clinic needs further investigation.

## Introduction

Chemotherapy-induced peripheral neuropathy (CINP) is a common side effect observed in 50% to 90% of cancer patients undergoing neurotoxic chemotherapy, with the added risk of becoming chronic in approximately 30% to 40% of cases^1^. Neurotoxic chemotherapeutic agents are closely linked to the development of CINP, and these agents include taxanes, vinca alkaloids, platinum-based drugs, Bortezomib, and thalidomide. Paclitaxel (PTX), a taxane, is the first microtubule-stabilizing drug discovered and represents a notable milestone in chemotherapy, being widely used for various cancer types^2–4^.

In the clinical setting, the prevalence of Paclitaxel-induced neuropathic pain ranges from 11 to 87%, with symptoms persisting in 67–80% of patients for up to one year after treatment ends^2^. Paclitaxel's mechanism of action relies on hyper-stabilizing microtubules, preventing the normal cycles of microtubule depolymerization and repolymerization within the cytoskeleton, making it an effective chemotherapy agent for tumor growth inhibition. Moreover, other mechanisms include mitochondrial dysfunction, immune responses, and calcium ion disruption, all of them conducting to peripheral neuropathy^5,6^. CINP symptoms significantly impact patients’ quality of life and include pain, tingling, cold-sensitivity, numbness and allodynia (increased sensitivity to non-painful stimuli) predominantly in the toes and fingers (“stocking and glove distribution”)^7^. Particularly noteworthy is the fact that effective symptom management often requires a Paclitaxel dose reduction to approximately 73.4% of the therapeutic dose, even though reductions below 85% are known to significantly decrease survival rates^8^.

The need for novel therapeutic approaches for CINP shifted the attention of both clinicians and researchers to new classes of drugs with different mechanisms compared to the standard therapy (tricyclic antidepressants, opioids, or anticonvulsants). The endocannabinoid system (ECS) plays a critical role in various physiological processes such as: inflammation, cognition, pregnancy, immunity, regulation of brain development, skin, respiratory and cardiovascular systems^9–12^. Modulating the ECS through the cannabinoid receptors (CBR) showed promising therapeutic effects in a wide range of diseases and pathological conditions, including CINP, making it a target for many drugs and therapies^13^.

Cannabinoids is an ‘umbrella term’ describing any substance that modulates cannabinoid receptors and produces similar effects to those produced by the Cannabis plant, regardless of its origin. Depending on their source, cannabinoids are classified into three major classes: endogenous synthetized cannabinoids, phytocannabinoids (PCs) and synthetic cannabinoids (SCs). The first discovered were PCs, the most known being Δ9-tetrahydrocannabinol (Δ9-THC) which was isolated from the *Cannabis Sativa* plant by R. Mechoulam in 1964^14^. Until 2012, more than 100 PCs were already described and isolated from a total of 545 phytocomponents of the Cannabis plant^14,15^. SCs are compounds functionally similar to PCs and were initially used to characterize the pharmacology of CBR^16^. SCs have significantly more affinity, up to 4 times higher for cannabinoid receptor 1 (CB1R) and 10 times higher for cannabinoid receptor 2 (CB2R), and as a consequence, may have greater side effects^17^. SCs were developed to better understand the mode of action of natural cannabinoids in the body and, by extension, the endocannabinoid system^16^. The study of some classes of synthetic compounds that modulate CB1R and CB2R started from the attempt to study pravadoline (an indole derivative), in an effort to find a non-steroidal compound with increased anti-inflammatory and analgesic effects. These studies led to the discovery of a number of structurally related compounds that interact with a G-coupled protein in the brain that inhibit adenylate cyclase and have antinociceptive actions. Thus, CB1R and then CB2R were identified and the actions of natural cannabinoids in the body were partially explained^18^. Further studies resulted in the development of a class of synthetic substances known as JWH, which contains over 100 compounds, all of which have a much higher affinity for the two receptors than natural cannabinoids^19^. JWH-182, a synthetic cannabinoid named after the chemist who synthesized a whole class of SCs, John W. Huffman, known chemically as 1-Pentyl-3-(4-propyl-1-naphthoyl) indole, is a naphthoyl-indole compound that exhibits a greater affinity for CB1R (Ki = 0.65 ± 0.003 nM) compared to CB2R (Ki = 1.3 ± 0.1 nM)^20,21^.

Despite SCs greater effects, more studies need to be conducted in order to distinguish between beneficial and life-threating effects of this class of cannabinoids. Also, we need to take into consideration the poorly understood pathogenesis of chronic neuropathic pain syndromes, the complexity of symptom expression, the absence of an ideal treatment and the attractive potential of the cannabinoids as new pain therapeuthic agents.

In the present study we aimed to establish the toxicity profile of synthetic cannabinoid, JWH-182, using both in vitro and in vivo tests in order to assess whether JWH-182 could be potentially efficient for palliating CINP. Furthermore, we wanted to develop and validate a fast extraction and LC–ESI–MS/MS method for quantitating the synthetic cannabinoid JWH-182 in mouse serum, in order to determine the best rate of administration for repeated administration toxicity evaluation.

## Results

### General in vitro toxicity of JWH-182

**Figure 1:**
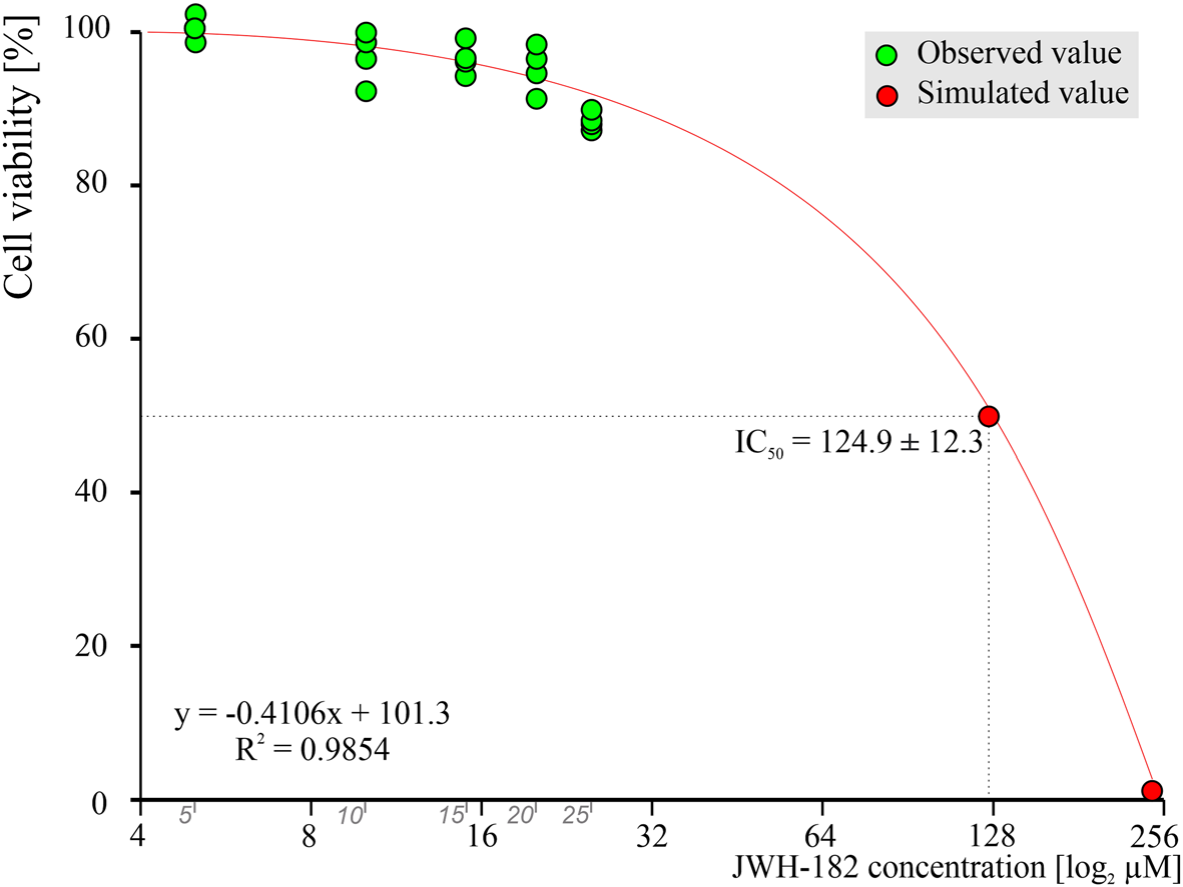
Concentration dependent cell viability of JWH-182 on fibroblast culture (V79 cell line). IC50 = 124.9 ± 12.3 µM.

Our results regarding the general toxic effects of JWH-182 suggest that even at the highest tested concentration, cell viability is > 80%, compared to the control. The concentration that is able to kill 50% of the cell population (IC 50 value) was estimated at 124.9 ± 12.3 µM (Fig. 1).

### In vitro neuroprotection of JWH-182

#### Characterization of DRG neuronal cells

Established primary dorsal root ganglion (DRG) neuron cultures were examined under a phase-contrast microscope at various time points (before treatment (T0), at 6, 24, 48, and 72 h after treatment administration) to analyze the overall cellular morphology and track the axonal length. At T0, all neuron’s somas appeared round, bright, and refractile, with a large nucleus. The neurons had long extended thin axons which connected and formed networks together.

#### In-vitro neuroprotection of JWH-182 in paclitaxel-induced neurotoxicity

**Figure 2:**
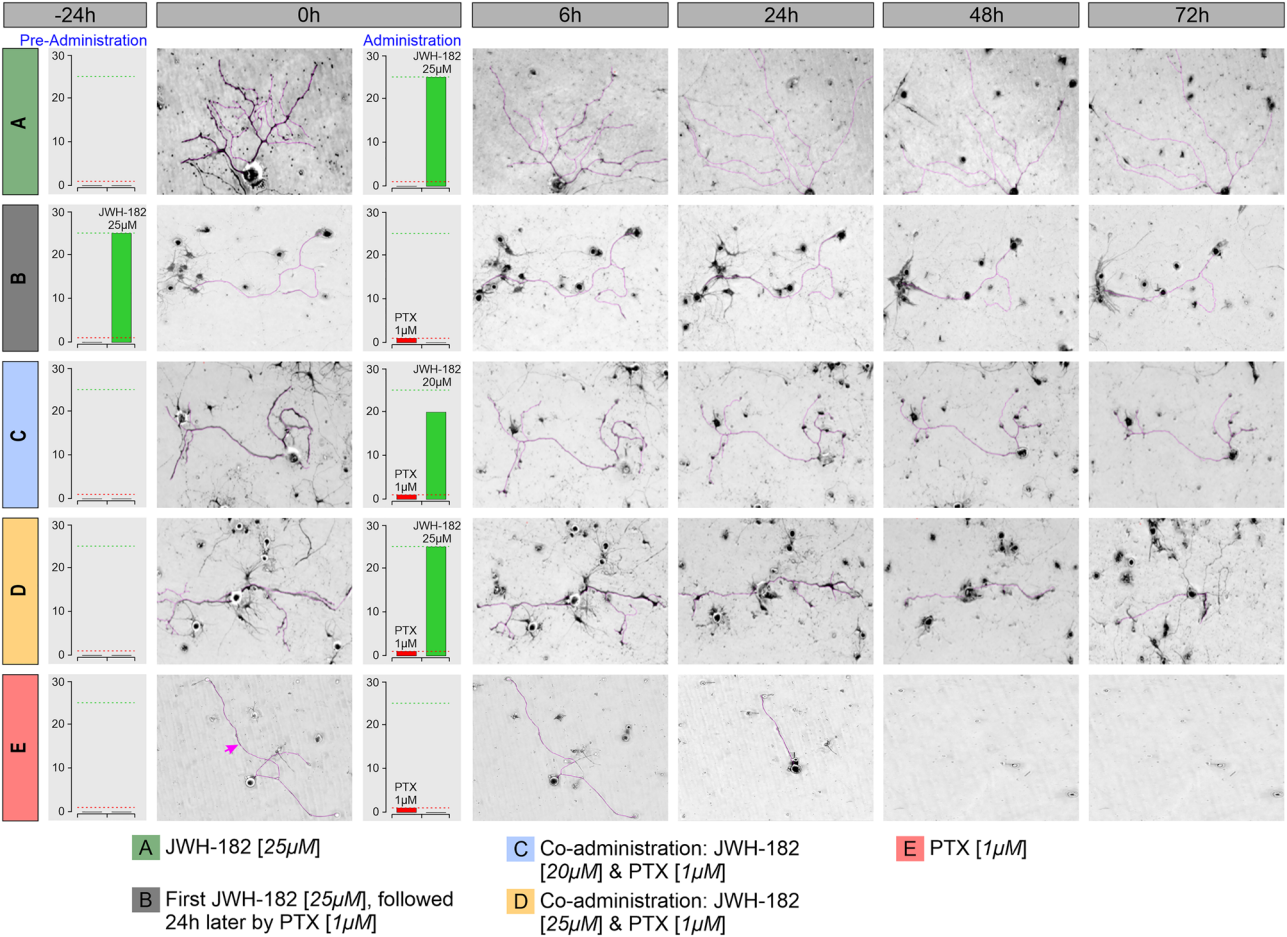
Time dependent axon outgrowth aspects (time-dynamic changes in cell viability and neurite length at different time points), measured with Image J (Neuron J plug-in). The pink arrow () indicates the axon aspect in the image.

For neurons treated only with PTX, axons seem to shorten progressively as early as 6 h post administration. At 72 h, we observed the total absence of axons and even cell death, regardless of the administered dose. The 10 µM concentration had the most toxic effects on the cells, at 24 h only 63% of the targeted neurons were still viable. On the other hand, for 0.1 µM and 0.5 µM, the neurotoxicity displayed a similar tendency, with axons’ length time-dependently decreasing in size and cellular death appearing for 13% and respectively 25% of the cells 48 h after therapy. When administering 1 µM PTX, only 25% of the cells were still viable at 48 h post therapy and had a maximal axon shortening (Fig. 2).

When administered in monotherapy (25 µM), JWH-182 clearly presented neuroprotective features on cultured neurons; axons seemed to be increasing in length (4–22%), compared to their initial dimensions at 6 h post-administration. The percentage of axonal shortening was significantly lower in comparison to the PTX-treated controls, with neurons still viable at 72 h post therapy (p < 0.0001).

The combination between JWH-182 and 1 µM Paclitaxel had significant protective effects on neurons at all investigated timepoints, compared to the PTX-only group (p < 0.0001). This effect was independent of the cannabinoid dose only for the first 24 h after administration. At 48 h and 72 h, we found that the axonal length was different in the case of neurons treated with 25 µM JWH-182 and 1 µM Paclitaxel as opposed to those treated with 20 µM JWH-182 and 1 µM Paclitaxel. The highest dose had a better protective role and decreased the axonal length with an average of 17% at 48 h and 34% at 72 h, compared to the lower dose, which shortened the axons on average by 33% and respectively 50% (p < 0.0001). Interestingly, at 48–72 h, neurons treated with 25 µM JWH-182 and PTX preserved their axons better than in the case of neurons treated with cannabinoids only (p < 0.1).

Neurons exposed first to 25 µM JWH-182, presented with an initial elongation of their axons. Obviously, 24 h later, after the addition of 1 µM PTX, the axonal lengths had a downward trajectory. However, at 72 h, the axons were still visible, with their lengths varying between 16–63% (p < 0.01), as opposed to PTX only treated neurons which were no longer viable.

**Figure 3:**
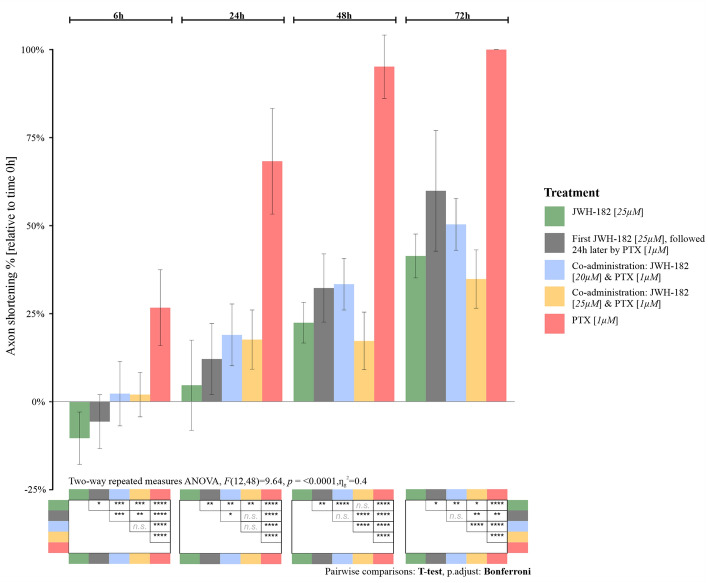
Effects of JWH-182 alone or in combination with Paclitaxel on axon outgrowth; the highest the score, the more neurotoxic the drug is.

If we were to identify which administration schedule conducted to a better neuroprotection, it seems that coadministration of 25 µM JWH-182 and 1 µM PTX had better results compared to pre & coadministration; at 48 h, the axonal lengths were between 62–92% compared to 51–82% (p < 0.0001). The same effects were also seen at 72 h (p < 0.01) (Fig. 3).

### Assessment of acute in vivo toxicity of JWH-182

**Figure 4:**
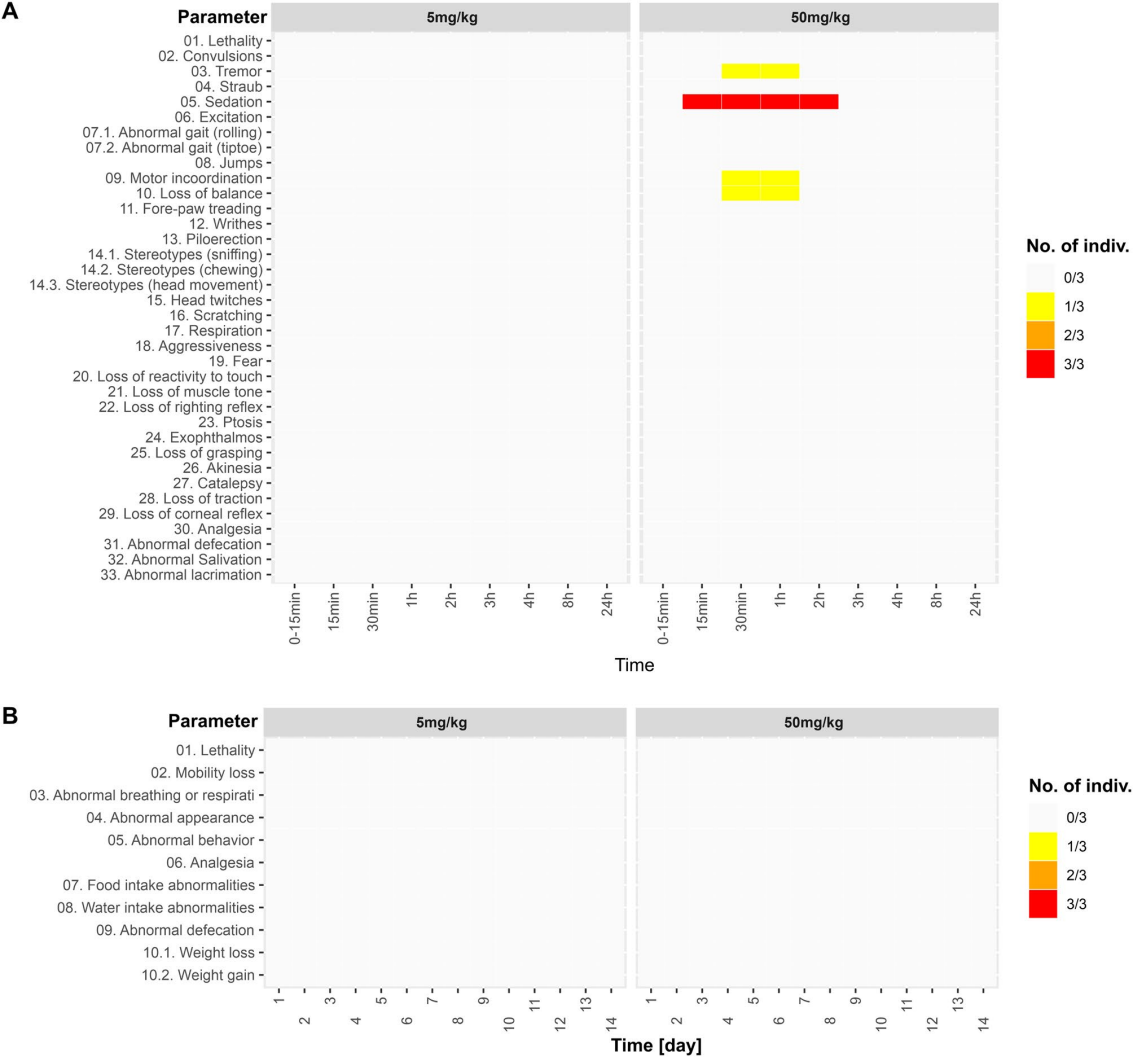
(**A**) Primary monitoring parameters during the first 24 h following JWH-182 intravenous administration. (**B**) General health parameters monitored for 14 days following JWH-182 intravenous administration.

In the first 24 h following administration, neither the control nor the 5 mg/kg groups showed any signs of toxicity. In the 50 mg/kg group, sedation, maintained for 24 h, was observed in all animals, loss of balance was observed in 2 animals, mild motor incoordination and tremor were observed in 1 animal (Fig. 4A). During the 14-day monitoring period, intraperitoneal administration of JWH-182 at doses up to 50 mg/kg had no observable toxic effect on general health. Furthermore, no significant deviation from the normal status was observed for general behavior, alertness state, food or water intake, or body weight variation (> 10%) in animals dosed up to 50 mg/kg (Fig. 4B).

### Quantitation of JWH-182 in mice serum

Six samples from mice to which doses of 5 mg/kg and 50 mg/kg were administered intraperitoneally 24 h before collection of blood samples were extracted and analyzed after confirmation of the accuracy and precision of the last set of calibrators. For 5 mg/kg, the results of serum dosing of JWH-182 using the method described in the Supplementary Material (“Introduction” section), were between 1.57 ng/mL and 3.31 ng/mL and for 50 mg/kg were between 16.05 ng/mL and 59.20 ng/mL. Results represented as the mean ± standard error of the mean are presented in the Supplementary Material (“Results” section) Fig. 4.

### Evaluating the antinociceptive potential of JWH-182

#### Evaluating the antinociceptive potential in the animal groups lacking neuropathic pain

Evaluating the antinociceptive impacts of JWH-182 entailed the administration of different doses, ranging from 5.00 to 0.63 mg/kg to groups of six animals. ED50 (mg/kg) which resulted for the studied nociceptive models can be found in Table 1. The outcomes derived from the administered dose sequences did not facilitate the determination of the ED50 value for the animal groups lacking painful neuropathy.

**Table 1:** Evaluating the antinociceptive potential of JWH 182 in animals without neuropathic pain.

Test	30 min	60 min	90 min	120 min
	ED50 mg/kg	ED50 mg/kg	ED50 mg/kg	ED50 mg/kg
Hot plate	4.774 ± 0.728 Y = 11.528 + 56.672*X R = 0.985 TCL (−4.478, 14.025)	2.588 ± 0.783 Y = 17.917 + 77.700*X R = 0.882 TCL (4.427, 1.410)	0.265 ± 0.230 Y = 62.699 + 22.041*X R = 0.936 TCL (0.575, 0.311)	0.991 ± 0.389 Y = 50.218 + 52.868*X R = 0.940 TCL (4.491, 1.525)
Tail Flick	4.348 ± 0.598 Y = 7.691 + 66.289*X R = 0.985 TCL (3.763, 1.250)	4.189 ± 0.466 Y = 13.243 + 59.080*X R = 0.990 TCL (3.478, 1.170)	4.719 ± 0.973 Y = −1.438 + 76.338*X R = 0.972 TCL (4.030, 1.336)	2.203 ± 0.202 Y = 5.281 + 130.39*X R = 0.988 TCL (4.978, 1.365)
Randall-Selitto	6.908 ± 3.116 Y = 96.384 + (−55.260)*X R = 0.932 TCL (1.400, 4.093)	16.719 ± 30.095 Y = 97.623 + (−38.933)*X R = 0.740 TCL (1.431, 4.243)	15.881 ± 21.180 Y = 94.140 + (−36.757)*X R = 0.823 TCL (1.439, 4.138)	14.507 ± 29.095 Y = 100.80 + (−43.733)*X R = 0.677 TCL (1.425, 4.305)

ED50 is calculated using Miller Tainter Method and is represented as the men ± standard error of the mean; TCL = True Confidence Limits.

#### Evaluating the antinociceptive potential of JWH 182 in animals with PTX-induced neuropathy

On both days 1 and 5 of the experiment, the animals did not display a reduction in the nociceptive threshold for either of the two tests, Hot Plate and Tail Flick. On day 10, the nociceptive threshold was reassessed, revealing a decrease of 124.8% for the Tail Flick test and 76.55% for the Hot Plate test. The reduction of the nociceptive threshold for day 18 of the experiment was 83.33% for the Hot Plate test and 122.33% for the Tail Flick test. The data confirms the onset of neuropathic pain starting with day 10 of the experiment, which is maintained throughout the experiment. The application of static mechanical stimulus led to a reduction in the nociceptive threshold, which decreased by 16.31% on day 5, 24.39% on day 10, and 26.28% on day 18 of the experiment. These results confirm the onset and maintenance of neuropathy.

The Tail Flick ED50 was evidenced 60 min post-administration of JWH-182 (ED50 = 2.627 mg/kg ± 0.093 mg/kg), while the Randall-Selitto ED50 was confirmed 30 min after administration (ED50 = 1.600 mg/kg ± 0.078 mg/kg) (Table 2). Notably, the compound exhibited greater potency in addressing mechanically induced nociception in comparison to thermal stimulus. There were no weight deviations recorded for the animal groups with PTX-induced neuropathic pain.

**Table 2:** Evaluating the antinociceptive potential of JWH 182 in the animal groups with neuropathic pain.

Test	30 min	60 min	90 min	120 min
	ED50 mg/kg	ED50 mg/kg	ED50 mg/kg	ED50 mg/kg
Hot plate	73.064 ± 188.31 Y = 7.096 + 23.021*X R = 0.798 TCL (3.754, 1.545)	8.655 ± 1.993 Y = 7.702 + 45.128*X R = 0.986 TCL (2.565, 1.224)	12.361 ± 7.740 Y = 29.322 + 18.935*X R = 0.938 TCL (3.658, 1.468)	1.944 ± 0.452 Y = 65.799 ± 54.729*X R = 0.936 TCL (1.421, 4.500)
Tail Flick	3.011 ± 0.184 Y = −13.574 + 132.81*X R = 0.995 TCL (4.318, 0.855)	2.627 ± 0.093* Y = 12.011 + 90.556*X R = 0.998 TCL (1.370, 5.933) (Ft:161.4 Fc: 259.68, α = 0.05)	1.752 ± 0.148 Y = 38.039 + 49.115*X R = 0.992 TCL (5.847, 1.67)	2.060 ± 0.230 Y = 7.287 + 136.10*X R = 0,983 TCL (4.827, 1.408)
Randall-Selitto	1.600 ± 0.078* Y = 32.888 + 83.829*X R = 0.998 TCL (0.074, 2.610) (Ft:161.4, Fc: 217.30, α = 0.05)	1.540 ± 0.450 Y = 36.173 + 73.780*X R = 0.931 TCL (4.506, 1.445)	1,224 ± 0,641 Y = 45.085 + 55.925*X R = 0.867 TCL (4.448, 1.444)	0.862 ± 0.415 Y = 52.774 + 43.019*X R = 0.929 TCL (4.454, 1.529)

ED50 is calculated using Miller Tainter Method and is represented as the men ± standard error of the mean; TCL = True Confidence Limits; α = Significance level; Ft = F table; Fc = F calculated.

Significant values are in bold.

### Repeated dose 28-day toxicity of JWH-182

Daily monitoring during the 4 weeks of the experiment of the two groups with intraperitoneal administration (control group—receiving vehicle solution, dosed group—receiving JWH-182 at dose 2.627 mg/kg) did not show significant changes regarding the animal’s general state of health. There were no deviations from the normal and no significant differences between the two groups in terms of behavior, state of alertness, water or food intake, nor in terms of body weight variation.

Regarding reactivity to stimuli, a slight increase in aggressiveness is observed in the group treated with JWH-182, but without statistical significance (Table 3).

**Table 3:** Sensory reactivity to stimuli tests results.

Group	Defecation	Urination	Ease of removal	Ease of handle	Approach response	Touch response	Tail pinch response
Control	0.21 ± 0.70	0.10 ± 0.10	0.10 ± 0.10	0.10 ± 0.10	0.10 ± 0.10	0.00 ± 0.00	0.10 ± 0.10
Dose	0.80 ± 0.35	0.30 ± 0.15	0.20 ± 0.13	0.00 ± 0.00	0.20 ± 0.13	0.30 ± 0.15	0.40 ± 0.16
p-value	0.8695	0.3006	0.5828	0.3681	0.5828	0.0767	0.1444
W	52.50	40.00	45.00	55.00	45.00	35.00	35.00

Wilcoxon rank sum test (independent samples) with continuity correction.

**Figure 5:**
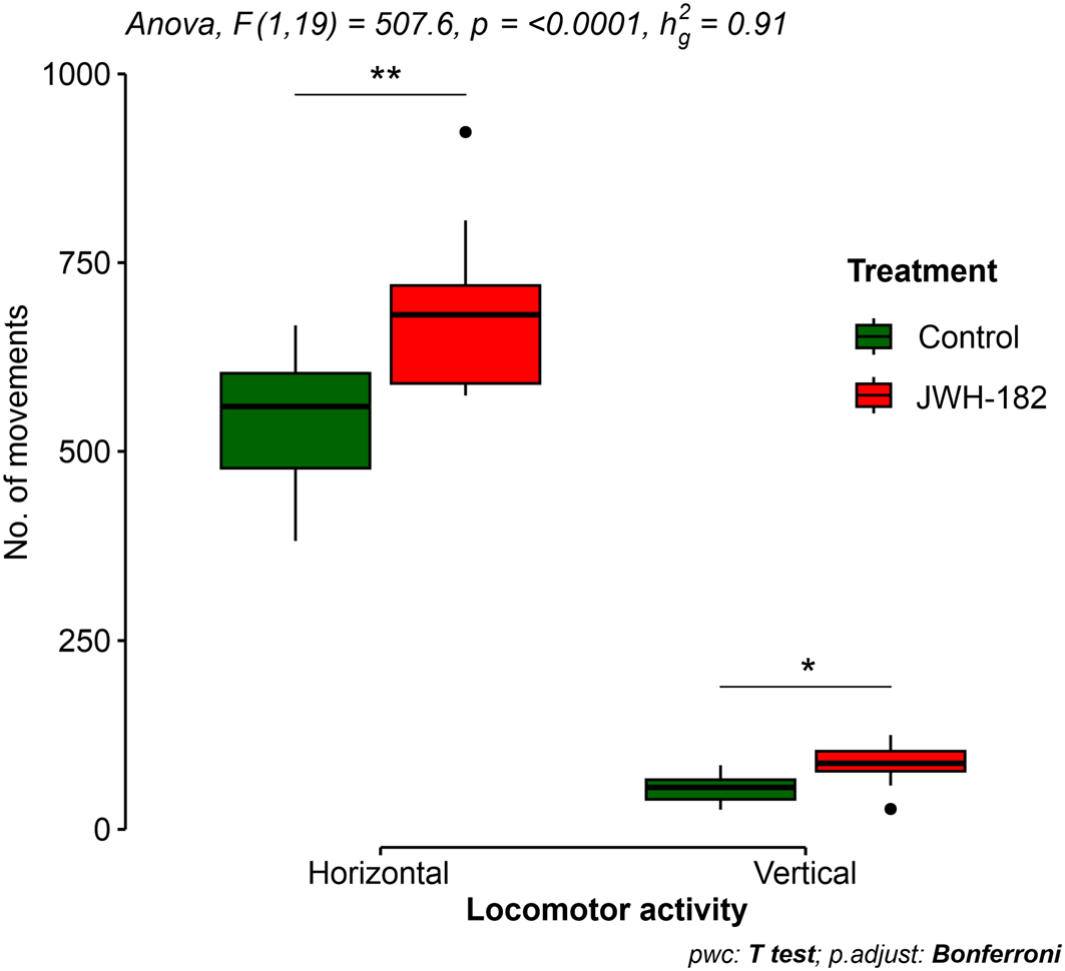
Horizontal and vertical (rearing) movements recorded for 10 min in the Activity Cage in the fourth week of administration period. Treated group presented a significant increase in spontaneous activity compared with control, both horizontally and vertically (p < 0.0001).

The motor activity evaluation revealed a significant increase in general activity in the JWH-182 treated group compared to the control group. Dosed animals were more active, with increased number of horizontal and vertical movements (Fig. 5).

### Gross necropsy and histopathology

External examination of the animals at the necropsy, after 14 days for acute toxicity study or after 28 days for repeated dose toxicity study, revealed no abnormalities in the mouth, nostrils, anus, coat, or limbs. Mice were well-conditioned, having a Body Condition Score (BCS) of 3^22^. No lesions or signs of disease were observed during internal examination. The histopathological screening of twenty-two organs revealed no significant histopathological abnormalities in the control (0 mg/kg) or experimental groups, regardless of the administered dose (see Supplementary Material, “Discussion” section, Fig. 5).

## Discussion

Recent research results shifted the attention of scientists towards in-depth exploration of the effects that SCs could have on a variety of diseases. Despite the relatively widespread use of these compounds as recreational drugs, scientific data on the acute toxic effects and long-term side effects of synthetic cannabinoids exposure remain mostly unknown. Most of the available data comes from multiple case reports on acute intoxications caused by accidental administration of toxic doses of substance mixtures, which include (SCs) among other compounds^23^. The toxicity profile of these substances seems to have some similarities to that of cannabis, although more serious adverse health effects are often seen with the former. In comparison to PCs, SCs have a higher risk of harm due to factors such as their full agonism at the CB1R and CB2R and their significantly higher potency, estimated to be 2–100 times more than 9-THC, based on in vitro studies^24^. With the exception of nabilone, no study has clearly documented the effects in humans or laboratory animals of long-term consumption of synthetic cannabinoids, which, unlike PCs, are not used as prescription medicines^25^. Despite the fact that some of the compounds have been found to have beneficial effects, the toxicity and unpredictable effects of synthetic cannabinoids are still an important aspect for the translation into the daily clinical practice. Among the most frequently reported pharmacological effects are sudden changes in blood pressure, convulsions, respiratory depression, stroke, or psychosis, thus thorough toxicity testing is a must^26^.

In the current study, the toxicity profile of the cannabinoid JWH-182 was found to be acceptable. The preliminary in vitro testing on fibroblast culture didn’t show any significant cytotoxicity compared to the controls. Following in vivo acute and repeated dose 28-day toxicity evaluation, our results highlighted that JWH-182 has observable toxic effects following acute administration only at 50 mg/kg, and no observable toxic effects in 28 days repeated administration at small doses.

After single-dose administration of 5 mg/kg JWH-182, no discernible alterations in general health parameters were observed, and there were no indications of stress or suffering, as evidenced by a final BCS of 3 after the 14-day monitoring period. All subjects in the 50 mg/kg group experienced sedation that lasted approximately 2 h after administration, and two animal experienced loss of balance. Also, one of the group members experienced tremors and mild motor incoordination during the first 2 h after administration. The tested compound is a strong agonist of CBR with an increased affinity for the CB1R^27^. In both, mice and humans, CB1R exhibit a notably higher prevalence in the central nervous system compared to CB2R receptors, with an overexpression observed in the basal ganglia cerebellum and peripheral nerves^28,29^. The overexpression of CB1R in these regions, that are pivotal for motor coordination can explain the appearance of some effects on the locomotor system^30^. Also, the modulation of stereotypes may involve CB2R on dopaminergic neurons, as suggested by Liu et al.^31^.

Repeated dose 28-day administration of a 2.627 mg/kg JWH-182 did not yield noteworthy alterations in behavior, alertness, general appearance, coat condition, or food and water intake when compared to the control group. Neither of the two groups exhibited indications of stress or suffering, maintaining a BCS of 3 until the end of the 28-day observation period. In the fourth week of administration, the experimental group exhibited a significant increase in motor activity as quantified through a 10-min test using the Activity Cage device, and an insignificant increase in aggressiveness in the case of touch and tail pinch response tests in comparison to the control group, emphasizing a behavioral perspective. The increased motor activity can be correlated with the abundance of CB1R, particularly in the output nuclei of the basal ganglia. These receptors play an important role in motor control, alongside influencing functions such as motor learning, behavioral responses, and emotional regulation^32,33^. Experimental evidence revealed that the activation of CB1R at this level by the administration of endocannabinoids elicits biphasic effects on locomotor function^34,35^. Substantially higher doses inhibit motor function, offering therapeutic utility in conditions marked by movement disorders like dystonia, tremors, or muscular dyskinesias^36^. Conversely, smaller doses enhance motor activity and induce a modest increase in aggressiveness, particularly observed in timid mice^34,37^.

Cannabinoids have been found to bring an improvement in case of chemotherapy-induced neuropathic pain^38^. Up to 97% of cancer patients treated with Paclitaxel develop peripheral neuropathy^39^. As Paclitaxel doesn’t cross the blood–brain–barrier, it specifically affects the peripheral system and leads to a predominantly sensory axonal neuropathy^40^. Although neurons are not dividing cells, they are also a target of Paclitaxel’s toxicity with DRG neurons highly susceptible to Paclitaxel accumulation^41^. Paclitaxel’s toxicity is not limited to the arrest of mitosis at G2/M via the microtubule stabilization, which conducts ultimately to apoptosis, but also includes dysregulation of the mitochondrion and calcium channel activity^40^.

According to the literature, CINP is a side effect of paclitaxel treatment that causes pain and numbness in the hands and feet^39,42^. The pathological mechanism of CINP is complex and not yet fully understood, but recent studies have shed light on some of the underlying mechanisms^43–45^. Here are some of the key findings: Paclitaxel accumulates in the dorsal root ganglia, which are clusters of sensory neurons located along the spinal cord; this accumulation can lead to damage of the sensory neurons and subsequent neuropathy^39^. CINP is associated with a length-dependent axonal sensory neuropathy, which means that the longest sensory nerves are affected first. This is thought to be due to the fact that these nerves have the highest energy requirements and are therefore more vulnerable to damage^39^; Paclitaxel can directly damage sensory neurons in the dorsal root ganglia, leading to axonal degeneration and cell death^7^. In vivo studies using rodent models have shown that paclitaxel can also have indirect effects on skin cells, immune cells, and glia, which can contribute to the development of CINP^43^; The are multiple other pathological mechanisms that contribute to the development of CINP, including oxidative stress, inflammation, and mitochondrial dysfunction^42^.

The exact mechanism for the neuropathic pain modulation by cannabinoids, is not totally understood. In broad terms, they act as ligands to the CBR in the central nervous system (mainly CBR1) and in the periphery (mainly CBR2). It is believed that the activation of CBR from primary afferent neurons, dorsal horn of the spinal cord and brain regions involved in pain processing is associated to a decrease in neuronal excitability and a dampening of neurotransmission^46^. It is also hypothesized that cannabinoids reduce alterations in cognitive and autonomic processing in chronic pain states^47^ targeting preferentially the affective features of pain due to the CBR distribution in the frontal-limbic area^48^. In addition, cannabinoids may attenuate low‐grade inflammation, another postulate for the pathogenesis of neuropathic pain^49^.

There is evidence from several studies that cannabinoids, such as cannabidiol (CBD) and Δ9-THC, can alleviate CINP without diminishing nervous system function or chemotherapy efficacy. In rats, a mixed CB1R and CB2R agonist suppressed nociception via a CB1R specific mechanism, as well as vincristine associated neuropathy via CB1R and CB2R activation^44^. Activation of CB2R suppresses nociception and central sensitization in a variety of tissue and nerve injury models of persistent pain. Neuropathic pain may also involve abnormal hyper-excitability in skin afferent nerves. Skin cells express CB2R and endothelin receptors, and when activated, release B-endorphin, which can reduce hyperalgesia mediated by pro-inflammatory pathways^44^. Although the anti-nociceptive effects of cannabinoids through the activation of CB1R on DRG neurons have been validated through site-specific drug administration and tissue-selective knockout, the primary site of CB2R-mediated antiallodynic effects remains uncertain.

Cannabinoids act via cannabinoid receptors, but they also affect the activities of many other receptors, ion channels, and enzymes. The mechanisms of the analgesic effect of cannabinoids include: inhibition of the release of neurotransmitters and neuropeptides from presynaptic nerve endings; modulation of postsynaptic neuron excitability; activation of descending inhibitory pain pathways; reduction of neural inflammation.

Shifting the focus on the direct effects that cannabinoids have on the axons of peripheral nerves, they can change the function of the neurons in several ways. By acting on the dendrites, they can interfere with the conduction of synaptic currents to the soma of the neuron. By acting in the soma, they can interfere with the generation of action potentials. By acting on ion channels in axon terminals, they can inhibit transmitter release from the terminals; the consequence is inhibition of neurotransmission with a presynaptic mechanism^50^.

In our study, we could indeed find a positive effect of the JWH-18 on neuronal culture in every tested scenario, compared to PTX-treated neurons. JWH-182 administered in monotherapy has been found to be neuroprotective in the first 24 h after administration, with axonal length increasing in size, in comparation to their initial size. Afterwards, the axonal length had a downward trajectory, tendency found also in the PTX-treated group, however the shortening was significantly lower.

It has been shown that cannabinoids are involved in Ca^2+^ regulation through mitochondria’s functions^51–53^. Mitochondrial calcium overload has been involved in the formation of the mitochondrial permeability transition pore (mPTP), which may further lead to the loss of mitochondrial function due to the perturbation of membrane potential and release of pro-apoptotic proteins into the cytosol. This can further conduct to modulation of peripheric neuron membrane excitability which can improve pain perception, but paradoxically, can also cause axonal degeneration, expressed by thinner and shorter axons^54,55^. A similar off-target mechanism of action has been found to be associated to PTX, its toxicity exerting not only on microtubules, but also on mitochondria and voltage-dependent calcium channels^40^. Interestingly in our study, when JWH-182 and PTX were simultaneously administered, the percentage of the axonal shortening was much lower compared to JWH-182 only group. This raises the question of whether there is a non-competitive antagonism between the cannabinoid and PTX at the axonal receptors, with JWH-182 decreasing the toxic potency of the chemotherapeutic drug. This feature seems to be applied only in case of co-administration, but not in pre & coadministration.

In case of animal models of chronic neuropathic pain, the administration of synthetic cannabinoid CP 55,940, a CB1R agonist, terminated thermal hyperalgesia and decreased mechanical allodynia, evaluated by hot plate test and von Frey test, respectively^56^. A single administration of WIN55, 212–2, a mixed CB1R/CB2R-receptor agonist, 7 days after nerve ligation, reduced cold allodynia and thermal hyperalgesia symptoms, evaluated by acetone and hot plate test, respectively^57^. The use of WIN55, 212–2 also improved mechanical allodynia at von Frey test in chemotherapy-induced chronic neuropathic pain, when animals presented behavior similar to those treated with opioids^58^. The combination of WIN55, 212–2 with the selective CB1R and CB2R antagonists SR141716 and SR144528 reversed allodynia improvement, evaluated by von Frey test, demonstrating that both cannabinoid receptors are directly involved in these mechanisms and can be targeted for treatment purposes^59^. Additionally, injection of JWH133 or JWH015, CB2R agonists, decreases mechanical allodynia after partial nerve ligation^56^. Moreover, endogenous cannabinoids such as Anandamide (AEA) and Palmitylethanolamide (PEA), may contribute synergistically to the control of pain transmission within the central nervous system, by attenuating the pain behaviour produced by chemical damage to cutaneous tissue^60^. Also, the addition of 2-Arachidonoylglycerol (2-AG) and AEA led to better sensorial behavior in neuropathic murine animals, increasing mechanical and thermal threshold as assessed by von Frey and hot plate tests^61,62^.

JWH-182 is a synthetic cannabinoid that activates the central CB1 receptor with a Ki value of 0.65 nM and the peripheral CB2 receptor with a Ki value of 1.2 nM^20,27^. Synthetic cannabinoids are functionally similar to Δ9-tetrahydrocannabinol (Δ9-THC), the major psychoactive substance in cannabis. The precise mechanisms by which JWH-182 interacts with the endocannabinoid system remain poorly understood^23^. However, it is known that JWH-182 activates the CB1R and CB2R, which are part of the endocannabinoid system^27^.

Based on the search results, JWH-182 has not been specifically studied in relation to paclitaxel-induced neuropathy. However, a study by Blanto et al.^63^ found that activation of the central CB2R system can prevent paclitaxel-induced neuropathy. Additionally, another study^64^ found that cannabinoids, including cannabidiol, can suppress neuropathic pain induced by chemotherapy drugs, including paclitaxel.

Our results demonstrated that on 3 experimental models of pain, 2 with thermal stimulus (hot-plate and tail-flick) and one with pressure stimulus (Randal-Selitto), for all animals with CINP, the cannabinoid was able to inhibit pain perception in a measurable way. However, there is no information available in the literature on how JWH-182 interacts with the Randall-Selitto test, and also there is no information available on how JWH-182 interacts with the tail Flick test.

Just like similar articles, our study emphasized that the medical use of cannabis is at an important tipping point in science^65^. However, there is an important need of more high-quality basic information, which would ensure the translation of the cannabinoids into the clinical trials for establishing the proper indications, contraindications, dosages, formulations, and data regarding specific patient groups.

Our study concludes that the novel synthetized cannabinoid JWH-182 has an acceptable toxicity profile in vitro, on fibroblast cultures, with no significant cytotoxicity compared to controls. In vivo acute and repeated dose toxicity evaluation revealed observable toxic effects after acute administration only at high doses, and no toxicity after 28 days of repeated administration at low doses. The compound exhibited a positive effect on neuronal cultures, providing neuroprotection in the first 24 h after administration and mitigating axonal length reduction over time. In animal models of CINP, the compound effectively inhibited pain perception in three experimental models, using both thermal and pressure stimuli. As such, JWH-182 can be considered an important candidate for addressing the significant unmet need of alleviating neuropathic pain. However, the translation of these effects to the human population needs further studies to endorse our findings.

## Materials and methods

### Drugs

Synthetic cannabinoid JWH-182 (Cayman Chemical, Ann Arbor, MI, USA) was dissolved in 0.9% saline and Polysorbate 80 (1%) and administered in volumes of 0.20 mL/10 g body weight (). Semisynthetic Paclitaxel was purchased from Sigma Aldrich (Darmstadt, Germany).

### Reagents

#### Drug formulation

Cremophor (surfactant) was purchased from Merck KGa (Darmstadt, Germany); Polysorbate 80 was purchased from Sigma-Aldrich (Saint Louis, USA) and 0.9% saline solution was obtained from B. Braun Pharmaceuticals S.A. (Timişoara, România), ethanol was purchased from Chimreactiv SRL (Bucuresti, România). For cellular studies, V79 cell line was purchased from ATCC (Virginia, USA); substrate 3-(4,5-dimethylthiazol-2-yl) 2,5 diphenyl tetrazolium bromide (MTT powder), Dimethylsulfoxide (DMSO), Dulbecco’s Modified Eagle Medium (DMEM) and Fetal Bovine Serum (FBS) were purchased from Sigma-Aldrich (Saint Louis, USA). For primary DRG neurons culture, dissection and culture medias (RPMI 1640, Neurobasal A), supplements (Penicillin–Streptomycin, B27, Glutamax), enzymes (Collagenase, Trypsin EDTA 0.25%, DNAse, Trypsin Inhibitor), Percoll, Poly-D-Lysine and Laminin were also purchased from Sigma-Aldrich (Saint Louis, USA).

#### Animal anesthesia

Isoflurane (inhalation vapour, liquid) was obtained from Laboratorios KARIZOO, S.A. (Caldes de Montbui, Spain).

#### Histopathology

All histological-grade tissue fixation, processing, embedding, and staining reagents were purchased from Richard-Allan Scientific (Kalamazoo, MI, USA) and used according to the specifications provided by the manufacturer.

### Animals

133 Swiss Albino mice, 8–12 weeks old, nulliparous and non-pregnant (females), 20–40 g weight were housed in the CEMEX animal research facility of the Grigore T. Popa University of Medicine and Pharmacy, Iasi, Romania, in a controlled environment (20 ± 4 °C room temperature, 50 ± 5% relative humidity, and a 12 h artificial light–dark cycle, 07:00 A.M./07:00 P.M.), in individually ventilated cages (IVCs), with ad libitum access to food and water. The experimental study was carried out in compliance with the ARRIVE guidelines, European Directive 2010/63/EU and AVMA Guidelines for the Euthanasia of Animals (2020)^66–68^, and it was authorized by the university's Research Ethics Committee (no. 47/17.02.2021) besides being approved by the Romanian National Sanitary Veterinary and Food Safety Authority (no. 34/07.04.2021). For intraperitoneal administration, single-use syringes with a division of 0.01 mL and needles with adequate size were used.

### General in vitro toxicity of JWH-182

General in vitro safety of JWH-182 was evaluated in case of fibroblasts culture using the MTT assay, a colorimetric method for evaluating cell viability that is sensitive, quantitative, and repeatable. The assay is focused on the ability of mitochondrial lactate dehydrogenase enzymes (LDH) in living cells to transform the water-soluble substrate 3-(4,5-dimethylthiazol-2-yl) 2,5 diphenyl tetrazolium bromide into a dark blue water-insoluble formazan. To dissolve the insoluble purple formazan product into a colored solution, a solubilization solution of dimethyl-sulfoxide is added.

V79 cells were incubated in 96-well plates (3,000 cells/well) for 24 h. Consequently, serial dilutions of each cannabinoid were administered (5 µM, 10 µM, 15 µM, 20 µM, 25 µM) and cells were exposed for another 24 h at 37 °C, 5% CO2. The dosages were chosen based on the literature^69,70^. Treated cell lines were examined microscopically to detect morphological changes or detached cells. Dead cells were washed once with Phosphate-buffered saline (pH 7.2 0.2) and residual live cells were stained with 0.5% MTT solution at a 110 μL/well concentration. Plates were incubated at 37 °C for 2 h, 90 µl MTT solution was discarded and 100 µl DMSO was used to dissolve newly formed intra-cytoplasmic MTT formazan crystals.

**Figure 6:**
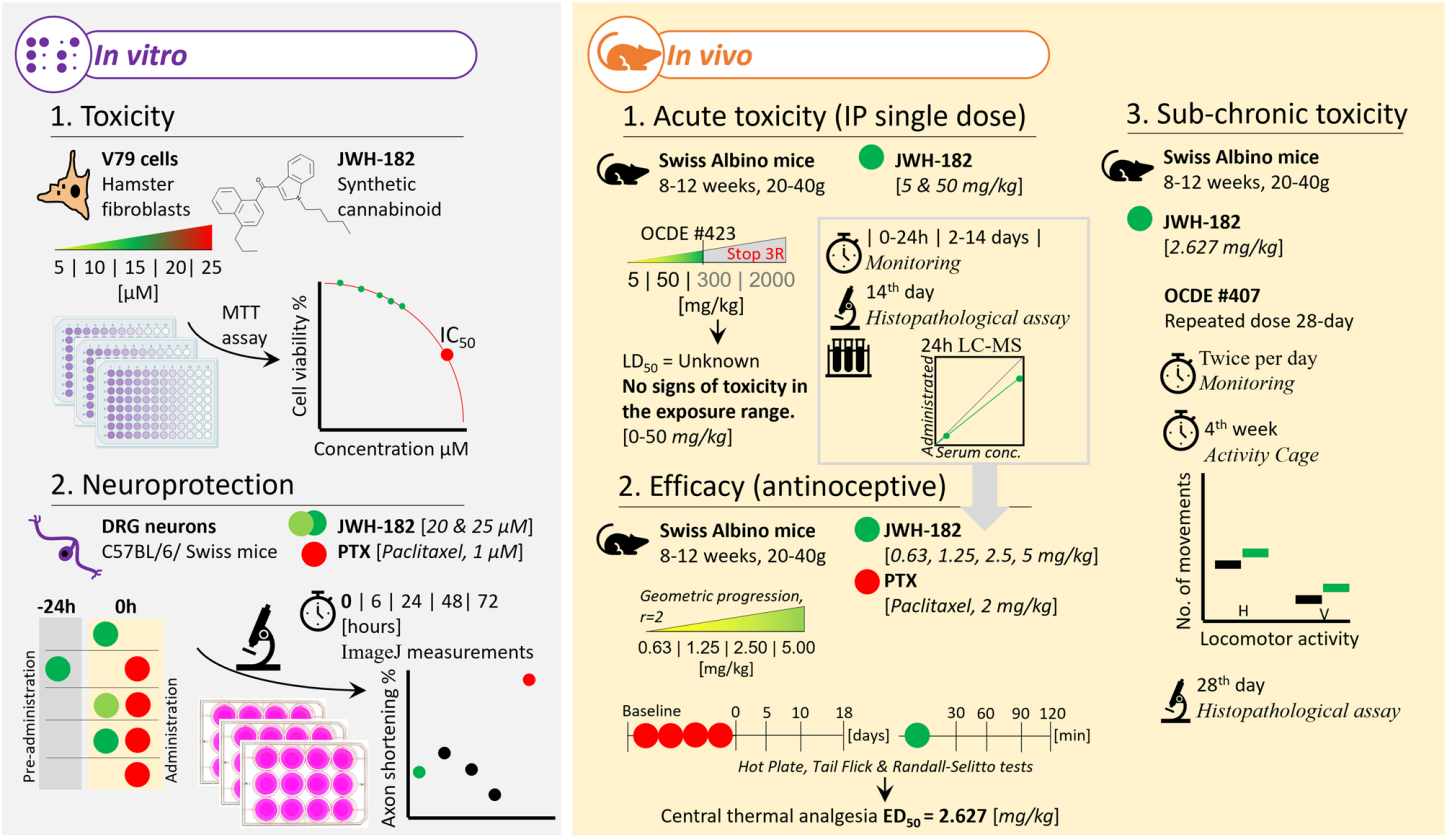
Graphical representation of the experimental design.

The experiment was conducted in triplicate. The viability of the cells was evaluated where optical densities were evaluated at 570 nm using a plate reader (EZ Read 400, Microplate Reader, Biochrom, UK). Control cells were cultured in normal media free of JWH-182. The viability percentage was calculated as follows: cell viability percentage = (OD of treated cells/OD of untreated cells) × 100. The IC50 was estimated using ED50 Plus v1.0 online software (National Institute of Respiratory Diseases, Mexico) (Fig. 6).

### In vitro neuroprotection of JWH-182

#### DRG neuron culture establishment

The ability of DRG explants to outgrow axons in vitro and to react by decreasing the axonal length when in contact with neurotoxic agents, represent a good, simple, and well-accepted model for studying peripheral neuropathy induced by antineoplastic agents^71^. The presented method is adapted by Öztürk et al.^72^. DRG tissues isolated from 6–8 weeks C57BL/6/Swiss mice. In brief, mice were deeply anesthetized with isoflurane by inhalation followed by scarification caused by anesthetic overdose. Under aseptic conditions, the vertebral column was isolated and spinal cord was exposed and scooped out. The vertebra bodies and adjacent ribs were carefully cleared from the muscles, fat and other surrounding tissues. The dissection pieces were placed on ice, and using a stereomicroscope, DRGs were localized, removed, collected from intervertebral foramina at both sides. The spinal nerve (immediately distal to the ganglion) was cut DRGs were placed in a sterile dish containing RPMI-1640 with 1% Antibiotic/Antimycotic solution. 15–20 isolated DRGs were enzymatically digested in the first enzymatic solution, which contained Collagenase 1 mg/ml for 45 min at 37 °C, 5% CO2. At the end of the incubation, collagenase was removed by washing 3 times gently with 1 ml of HBSS. Afterwards, washed DRGs are incubated in Trypsin EDTA 0.25% for 15 min at 37 °C, 5% CO2. 10 µl DNAse is added to the same tube for the third enzymatic reaction, and DRGs are gradually triturated for approximative 10 min with 1000 µl pipette tip, 200 µl pipette tip and finally with insulin needle, until the tissue is homogenized, followed by incubation for 30 min at 37 °C 5% CO2 for 30 min. At the end of the incubation period, Trypsin–EDTA and DNAse are removed by centrifugation at 180*g* for 3 min. Cells are resuspended in Trypsin inhibitor solution added to the top of Percoll gradient gently and centrifuged at 530*g* for 20 min. The DRG neurons are collected between the layers of the Percoll gradient, followed by washing by adding culture medium and centrifugation at 180*g* for 3 min. 12-well plates were pre-coated with 1 mg/mL Poly-D-lysine and 0.1 mg/mL laminin for 2 h or overnight in 4 °C, then washed one time with distilled H_2_O directly before seeding the cells in culture medium. DRG neuronal cells were then pre-seeded onto the center of the coated coverslips for 2 h in an incubator with 37 °C and 5% CO_2_. Afterwards, warm culture media was gently added to the wells, and the cells were maintained again at 37 °C with 5% CO_2_. The growth and morphology of neurons were monitored after 24, 48, and 72 h to detect the suitable time of treatment.

#### Axon tracing using ImageJ

Neurotoxicity of the treatments was assessed by the measurement of axon length by using Axon Tracer, a plugin for Image J. Briefly, a sample image pair from cultured DRG neurons were opened in Image J (v1.46r (National Institutes of Health, Laboratory for Optical and Computational Instrumentation, University of Wisconsin, Madison, WI, USA) subtracted the background, converted to 8-bit grayscale and then individually opened in axon tracer plugin. Thereafter, the resulting images were inserted into the axon tracer, with the threshold adjusted manually before starting the automated tracing. The results were normalized with those from the control group.

#### Axon preservation efficacy tests

The tests were divided in 4 scenarios. (1) Determination of the dose of PTX that induced the maximal shortening in the axonal length and not cell death, in 4 different dosages; (2) assessment of the effects of JWH-182 administered in monotherapy; (3) assessment of the effects of JWH-182 administered simultaneously with Paclitaxel in two different dosages; (4) pretreatment with JWH-182 for 24 h, and the addition of PTX after. For each scenario, DRG neurons were seeded in 12-well plates, cultured at 37 °C, 5% CO2 for 48 h until most axons outgrew. Consequently, images of axons were obtained at T0, after that treatments were administered according to the testing group.

For the first scenario (neurotoxic PTX dose identification), neurons were treated with different concentrations (100 nM, 500 nM, 1 µM, 10 µM), selected based on the existing literature^73–75^. For the second scenario (JWH-182 monotherapy), neurons were treated with 25 µM, dose chosen according to the previously mentioned toxicity tests, in line with the existing literature^76–78^. Images of the same neurons identified at T0 were take 6 h, 24 h, 48 h and 72 h after the treatment. After the preliminary tests regarding general JWH-182 effects on neurons and the identification of the PTX dosage that induces the maximal neurotoxic effect on axons, but with minimal cellular death, we proceeded with the next stages. For the effects of co-administration of the cannabinoid and PTX (scenario 3), fully matured neurons were simultaneously treated with a combination of either 20 µM JWH-182 and 1 µM Paclitaxel or 25 µM JWH-182 and 1 µM Paclitaxel and incubated at 37 °C, 5% CO2; images of the axons were obtained before any treatment and 6 h, 24 h, 48 h, 72 h after administration. For the last scenario, fully developed neurons were pre- treated with 25 µM JWH-182, incubated for 24 h at 37 °C, 5% CO2. 1 µM Paclitaxel was further added on top the cannabinoid-treated neurons, and images of of the axons were obtained again, at the same timepoints. The reference controls were: DRG neurons cultured in normal growth media and neurons treated with only 1 µM Paclitaxel. The growth media of the controls contained the similar concentration of DMSO (0.1%) used for treatment formulation, in order to exclude any solvent effects on cell viability. All experiments were performed in triplicate.

#### Statistical analysis

To further investigate the treatments' significance on axon length, a two-way ANOVA analysis was performed, followed by pairwise comparisons using T-tests. The normality of the data was evaluated using the Shapiro–Wilk Normality Test, and the Levene's test for homogeneity of variances was conducted. To address the issue of multiple comparisons, Bonferroni adjustment was applied to the p-values (Fig. 6).

### Assessment of acute in vivo toxicity of JWH-182

The acute in vivo toxicity study for intraperitoneal administration was carried out starting from OECD 423: Guideline for Chemical Testing—Acute Oral Toxicity^79^. This method can be used to assess the median lethal oral dose (LD50) and classify the substance, plant, or mixture of substances in the Globally Harmonized Classification System for Chemical Substances and Mixtures (GHS). The method was corroborated with the European Medicines Agency's ICH guideline M3(R2) on non-clinical safety studies for the conduct of human clinical trials and marketing authorization for pharmaceuticals, which specifies that lethality should not be an intended endpoint in studies assessing acute toxicity. Thus, the administration was discontinued at the 50 mg/kg dose, when enough information about the toxicity and observed adverse events for JWH-182 was obtained. The primary monitoring protocol and parameters were adapted from the Primary Observation (Irwin) Test in Rodents^80^. After receiving a single dose of JWH-182, each individual was monitored continuously for the first 24 h, monitoring toxicity parameters such as sedation, convulsions, motor incoordination and tremors, using a video-tracking system, and once a day, recording general health parameters, for the next 13 days, as described previously by our group^81^ (Fig. 6).

### Quantitation of JWH-182 by LCMS

For method development and validation neat rat serum, obtained from blood collected by cardiac puncture, was used. Six blood samples from mice treated with JWH-182 were collected by retro‐orbital sampling at 24 h after compound administration. After blood collection, the tubes were maintained for 30 min in vertical position and the serum was separated from the clot by centrifuging for 10 min at 2000×*g*. The separated serum was transferred in clean labelled 1.5 mL polypropylene tubes and the tubes were frozen at −24 °C.

The methodology for synthetic cannabinoid extraction from mouse serum and LC–ESI–MS/MS analysis was largely described in the supplementary material.

### Evaluating the antinociceptive potential of JWH-182

JWH-182 was tested for nociceptive sensitivity and its antinociceptive potential on groups of animals in the presence and absence of neuropathic pain. The Hot Plate test (HP) and the Tail Flick test (TF) were used as thermal stimulation nociception models, and the Randall-Selitto (RS) test was used as mechanical stimulation nociception model (Fig. 6).

#### Evaluating the antinociceptive potential in the animal groups without neuropathic pain

Antinociceptive action of JWH-182 was evaluated on groups of 6 mice (weighing 30–40 g), which received doses of 0.63–1.25–5.00 mg/kg intraperitoneally. The dosage sequence was established according to the acute in vivo toxicity assessment and the compound remaining in the blood serum, 24 h after the administration^82^.

#### Evaluating the antinociceptive potential in the animal groups with neuropathic pain

In order to induce neuropathic pain mice received a daily dose of 2 mg/kg intraperitoneally for 4 straight days, in saline:Cremophor EL:ethanol (99:0.5:0.5)^83–86^. The nociceptive threshold was evaluated on first day before administering Paclitaxel, as well as on days 5, 10, and 18. The reduction in nociceptive threshold by at least 20% compared to the initial testing and the control group for any of the applied stimuli suggests that neuropathy was installed^87^.

#### Testing nociceptive sensitivity

(A) The Hot-Plate test was performed using the Woolfe and Macdonald (1944) method, modified by Eddy and Laborit (1953), with a Ugo Basile 7280 device. ^88^. The animal was placed on the heated metal surface at 52.5 ± 0.1 °C and repeatedly exposed to the thermal stimulus 45 min before and 30, 60, 90, and 120 min after the test substance administration. The pain response (licking the posterior paws, jumping, shaking the posterior paws) latency was assessed (cut-off time of 30 s). Collected data was expressed as a percentage related to the Maximum Possible Effect (% MPE), through the following formula:

$${{\%}}\;{\text{Inhibition}} = \left[ {\left( {{\text{Tx}} - {\text{T}}0} \right)/\left( {{\text{Tm}} - {\text{T}}0} \right)} \right] \times 100,$$

where T0 = response latency prior to administration of JWH-182; Tx = response latency at x minutes after administration; Tm = cut-off time.

(B) The Tail Flick test was performed using the Ugo Basile no. 7360/05998 device, after the method of Ben-Bassat (1959), modified by Grotto and Sulman (1967)^88–91^. A thermal stimulus (radiant heat, 55 ± 0.1 °C) was applied on the middle part of the animals’ tail and the pain response (tail retraction) latency was assessed (cut-off time of 10 s). The nociceptive sensitivity of the animals was pretested 45 min before the experiment^83,92^. Data was expressed in the same way as for Hot Plate test.

(C) The Randall-Selitto test consists of applying a static mechanical stimulus with a progressively increasing force on the hind paw of the animal. The paw withdrawal is the animal's nociceptive response^93,94^. With this test, mechanical allodynia and mechanical hyperalgesia can be assessed. We used Ugo Basile no. 37215 analgesimeter device, with a progressively increasing force of 16 g/s (cut-off = 15.62 s)^95,96^. Pretesting the animals consisted of successive tests, with a 1–2 min pause, starting with the left paw, then with the contralateral paw. Three tests for each of the hind paws were done, for which the mean of the results was calculated. For assessing the antinociceptive action of the studied compound, the pressure stimulus was applied before and after administering the substance at 30, 60, 90, 120 min.

Data was expressed as a percentage related to % MPE, through the following formula:

$${{\%}}\;{\text{Inhibition}} = \left[ {\left( {{\text{g}}_{{\text{x}}} - {\text{g}}_{0} } \right)/\left( {{\text{g}}_{{\text{m}}} - {\text{g}}_{0} } \right)} \right] \times 100$$

where g_0_ = response latency prior to administration of JWH-182; g_x_ = latency at different time intervals following the administration; g_m_ = cut-off weight.

#### Statistical analysis

ED50 of JWH-182 was determinate using the Miller-Tainter method. The statistical parameters of the regression lines (ANOVA) highlight the significance of the obtained data^97,98^. A statistically significant difference between the compared groups was considered for p < 0.05. The results are represented as the mean ± standard error of the mean.

### Repeated dose 28-day toxicity of JWH-182

The repeated dose 28-day toxicity study was carried out based on the OECD 407 Guidelines^99^. 20 Swiss Albino mice were randomly assigned in two treatment groups of 5 male and 5 female, one group receiving the vehicle solution (NaCl 0.9% with 1% Polysorbate 80) and the second group receiving JWH-182 at dose level of 2.627 mg/kg. The doses were chosen based on the results of the acute toxicity and efficacy studies. Throughout the study period, all animals were visually examined twice daily for clinical signs of toxicity, morbidity, and mortality, both before and after administration. For each animal in the study, a complete monitoring sheet was filled in with detailed clinical observations. The motor activity was determined, during the fourth exposure week, in order to assess the effect on the central nervous system by recording the horizontal and vertical movements of mice in the Activity Cage Ugo Basile by positioning the animal in the center of the device while recording its movements for 10 min^100^. Sensory reactivity to stimuli were determined by carrying out five tests, each having two possible results (0—normal response, 1—escape or defense reaction)^99^. Data from reactivity to stimuli assay were analyzed using Wilcoxon rank sum test (independent samples) with continuity correction. Prior to this, the normality of the data was evaluated using the Shapiro–Wilk Normality Test. Data regarding motor activity were statistically processed using ANOVA, after the Shapiro–Wilk Normality Test and Levene's test for homogeneity. Additionally, a pairwise comparison using T-test, followed by the adjustment of the p-value by Bonferroni was conducted. Every animal was weighed once a week. Food consumption was measured on a weekly basis (Fig. 6).

### Gross necropsy and histopathology

A full necropsy was carried out on all individuals involved in acute and repeated dose toxicity studies following lethal injection, involving a careful examination of the external surface of the body, all openings, internal cavities and their contents. After gross necropsy, twenty-two internal organs were collected and histopathological analyzed as previously described by our team^81^ (Fig. 6).

### Institutional review board statement

The experimental study was performed in accordance with the European Directive 2010/63/EU and has been approved by the university’s Research Ethics Committee (No. 47/17.02.2021) and authorized by Romanian National Sanitary Veterinary and Food Safety Authority (No. 34/07.04.2021).

### Supplementary Information

Supplementary Information.

## Data Availability

The datasets used and/or analyzed during the current study are available from the corresponding author upon reasonable request.
