# Supplementary material for: JWH-182: a safe and effective synthetic cannabinoid for chemotherapy-induced neuropathic pain in preclinical models
Source: Sci Rep. 2024 Jul 15;14:16242. doi: 10.1038/s41598-024-67154-y (PMC11247095; doi:10.1038/s41598-024-67154-y)
Supplement: Supplementary file 1 — Supplementary Information. [file 41598_2024_67154_MOESM1_ESM.docx]

**Supplementary material**

**JWH-182: a safe and effective synthetic cannabinoid for chemotherapy-induced neuropathic pain in preclinical models**

Leontina-Elena Filipiuc^1,2,#^, Ioana Creangă-Murariu^1,2,#^, Bogdan-Ionel Tamba^1,2,*^, Daniela-Carmen Ababei^1,3^, Răzvan-Nicolae Rusu^3^, Gabriela-Dumitrița Stanciu^1^, Raluca Ștefanescu^1^, Mitică Ciorpac^1^, Andrei Szilagyi^1^, Raluca Gogu^1^, Silviu-Iulian Filipiuc^1^, Ivona-Maria Tudorancea^1,2^, Carmen Solcan^4^, Teodora Alexa-Stratulat^2^, Marinela-Carmen Cumpăt^5,6^, Doina-Clementina Cojocaru^5,6^ and Veronica Bild^1, 3, 7^

**1. Methodology for Synthetic Cannabinoid Extraction from Mouse Serum and LC-ESI-MS/MS Analysis**

The quantification of JWH-182 using liquid chromatography-mass spectrometry (LCMS) has been previously documented for herbal products and whole human blood samples [1–3].

**1. Reagents**

The standards JWH-182 and JWH-018-d9 were purchased from Cayman Chemical in crystalline form. Acetonitrile was purchased from VWR Chemicals (Rosny-sous-Bois, France) and was of HPLC, UHPLC and LC-MS grade. Methanol was purchased from LAB-SCAN Analytical Sciences (Gliwice, Poland) and was of HPLC grade. Formic acid 99.7% was from Merck (Darmstadt, Germany). Ultrapure water (0.055 μS/cm c) was produced by an Arium Mini Plus UV system (Sartorius Lab Instruments Gmbh & Co., Goettingen, Germany).

**2. Sample collection for LC-ESI-MS/MS**

For method development and validation neat rat serum was used. Rat blood was obtained from healthy adult rats age 8-12 weeks. The blood was collected by cardiac puncture in 1.5 mL microcentrifuge tubes. After blood collection, the tubes were maintained for 30 minutes in vertical position and the serum was separated from the clot by centrifuging for 10 minutes at 2000 × g. The separated serum was transferred in clean labelled 1.5 mL polypropylene tubes and the tubes were frozen at -24 ºC.

Six blood samples from mice treated with JWH-182 were collected at 24 hours after administration of the synthetic cannabinoid. The blood was collected by retro‐orbital sampling and the serum was separated and preserved as described above for rat serum.

**3. Methodology for synthetic cannabinoid extraction from mouse serum**

First, 40 μL of mouse serum were mixed with 10 μL water: methanol (1:1) and 10 μL internal standard (40 ng/mL of JWH-018-d9) diluted in water: methanol (1:1). Proteins were precipitated by adding 80 μL ice-cold acetonitrile to each sample followed by mixing in a vortex mixer for 2 min at 6 ºC, 1000 rpm and centrifugation at 4000 rpm for 10 min at 4 ºC. Next,100 μL of the supernatant were added to 100 μL ultrapure water in a second 1.5 mL microcentrifuge tube. The tube was centrifuged at 4000 rpm for 10 min at 4 ºC. The supernatant (100 μL) was transferred to a clean 1.5 mL microcentrifuge tube and placed in ice until LC-ESI-MS/MS analysis. Ten microliters of the sample were injected in the separation column.

**4. Preparation of standard stock solutions and calibrators**

Stock solutions of each of the standards, JWH-182 and JWH-018-d9 were prepared at the concentration 1 mg/mL in methanol. Daily, working solutions were prepared in methanol: water (1:1) at the following concentrations 20000, 4000, 2000, 800, 400, 200, 80, 40, 20, 8, 4, 2, 0.8, 0.4, 0.2 ng/mL for JWH-182 and 12000, 120 and 40 ng/mL for JWH-018-d9. The standard working solutions were used for spiking neat rat serum samples producing calibrators containing 1000, 500, 200, 100, 50, 20, 10, 5, 2, 1, 0.5, 0.2, 0.1, 0.05 ng/mL serum of JWH-182 and 10 ng/mL serum of internal standard.

**5. LC-ESI-MS/MS analysis**

A Kinetex C18 (100 × 4.6 mm, 2.6 μm,100 Å) HPLC separation column (Phenomenex, Torrance, CA, USA) was used in the column compartment of an Agilent 1200 HPLC coupled to an Agilent 6410 electrospray-triple quadrupole mass spectrometer. A 10:1 post-column splitter was connected. The mobile phases were A (0.1% formic acid in water) and B (0.1% formic acid in acetonitrile). At the beginning of the gradient the mobile phase B was held constant at 50% for 1 min. A linear increase from 50% B to 90% B for 3 min was selected and was held constant at 90% B for 6 min. Next, mobile phase B decreased to 50% in 1 min and the separation column was equilibrated at 50% B for 5 min. The flow rate was 0.8 mL, and the column temperature was 24 ºC. The mass spectrometer was operated in positive mode. The source parameters were a capillary voltage of 4000 V, a gas of temperature 325 ºC, a gas flow of 8 L/min, and a nebulizer of 35 psi.

**Table 1.** The parameters of the mass spectrometer for each compound and the retention time.

| **Chemical compound** | **Precursor ion (m/z)** | **Product ion (m/z)** | **Dwell time (ms)** | **Fragmentor (V)** | **Collision**  **Energy (V)** | **Retention time (min)** |
| --- | --- | --- | --- | --- | --- | --- |
| JWH-182 | 384.3 | 197.1 | 100 | 135 | 22 | 8.19 |
| JWH-018-d9 | 351.2 | 155.0 | 100 | 135 | 25 | 6.87 |

**6. Method validation**

Selectivity: Three blank rat samples were spiked with internal standard (zero sample) and were analyzed for interfering peaks.

Limit-of-detection (LOD): The LOD was estimated using the last concentrations from the calibration curve based on the signal-to-noise ratio of 3 calculated by Mass Hunter taking into consideration the peak-to-peak approach (Fig. 1).


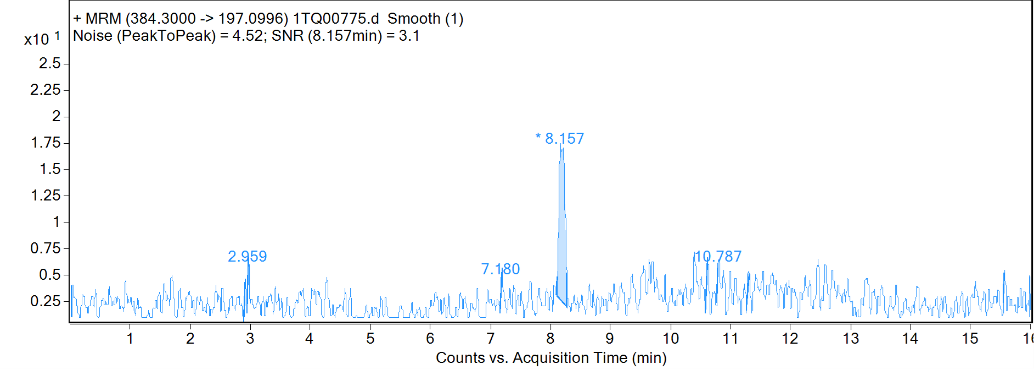


**Figure 1.** Extracted chromatogram for the transition m/z 384 → 197.1. A sample of serum spiked with 0.2 ng/mL JWH-182 was extracted and analyzed by LC-ESI-MS/MS.

Linearity of calibration: Eleven calibrators were analyzed in the range 0.5-1000 ng/mL in three replicates. Calibration curves were obtained for the high concentration range and low concentration range by linear regression (Table 2). LOQ was estimated at 0.5 ng/mL based on the linearity, accuracy, and precision of the calibrators.

***Table 2.*** *Accuracy and precision data for the calibration curves.*

| **Calibrator concentration (ng/mL ser)** | **High concentrations range (20-1000 ng/mL)** | | **Low concentrations range (0.5-50 ng/mL)** | | **Precision CV% (inter-run, n=3)** |
| --- | --- | --- | --- | --- | --- |
|  | **Linearity** | **Accuracy** | **Linearity** | **Accuracy** |  |
| 1000 | y=0.1624x-0.2588  R^2^ = 1 | 99.8 |  |  | 6.8 |
| 500 |  | 100.6 |  |  | 8.7 |
| 200 |  | 98.2 |  |  | 0.9 |
| 100 |  | 98.7 |  |  | 1.7 |
| 50 |  | 101.6 | y=0.1603x-0.0036  R^2^ = 0.9998 | 99.7 | 0.8 |
| 20 |  | 107.8 |  | 101.2 | 3.7 |
| 10 |  |  |  | 103.5 | 2.4 |
| 5 |  |  |  | 92.9 | 3.4 |
| 2 |  |  |  | 96.6 | 4.8 |
| 1 |  |  |  | 88.3 | 6.9 |
| 0.5 |  |  |  | 114.2 | 8.7 |

Carryover was investigated through the analysis of methanol: water (1:1) after a sample spiked with 1000 ng/mL JWH-182 and subjected to the extraction procedure. Carryover effect was not seen at this concentration.

**7. Matrix effect, recovery and process efficiency**

Matrix effect, recovery and process efficiency were calculated for the synthetic cannabinoid JWH-182 spiked at 4 concentrations 1, 10, 100 and 1000 ng/mL. Three sets of samples were prepared. In set A a sample of 40 μL of water was spiked with standard and was subjected to the complete extraction procedure. In set B blank rat samples were spiked post-extraction and in set C blank rat serum was spiked before the extraction procedure. Matrix effect, recovery and process efficiency were calculated according to the following equations which were reported previously [4]:

ME (%) = B/A×100;

RE (%) = C/B ×100;

PE (%) = C/A ×100 = (ME ×RE)/100.

**8. LC-ESI-MS/MS method optimization**

The instrumentation, the precipitation procedure, the separation column and gradient described previously for 11 natural cannabinoids by Filipiuc et. al. were tested and provided good extraction and chromatographic separation for JWH-182 and JWH-018-d9 [5]. The LC-ESI-MS/MS run of 16 minutes ensures fast analysis of the samples and provides equilibration of the separation column prior to the next analysis. The separation column is equilibrated at 50% B and the sample is diluted before the last centrifugation step with 100 microliters of ultrapure water which reduces the acetonitrile in the sample to less than 50% increasing the binding to the chromatographic material.

For the exclusion of the fragmentation which may occur in the electrospray source due to fragmentor voltage, ten microliters of 1000 ng/mL JWH-182 in methanol: water (1:1) were injected in the separation column and the mass spectrum obtained at the retention time of 8.19 m/s was evaluated. According to the results, at the fragmentor voltage of 135 only intact protonated molecule was observed.

The same solution was used to optimize further the collision energy for achieving maximum amount of the product ion at m/z 197.1. For this purpose, the collision energy was varied at 0, 5, 10, 15, 20, 25, 22 and 24 V (Figure 2.).


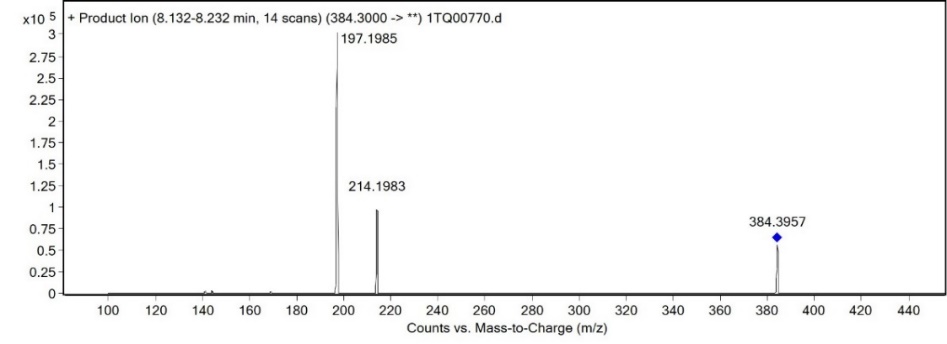


**Figure 2.** Mass spectrum of the product ions resulting at CE 22.

| (a) | 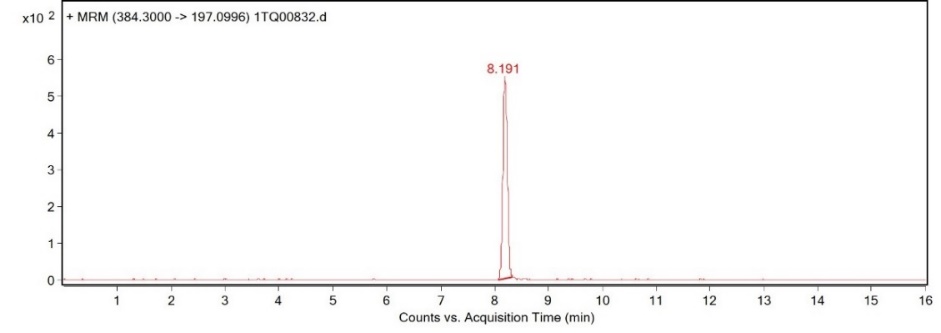 |
| --- | --- |
| (b) | 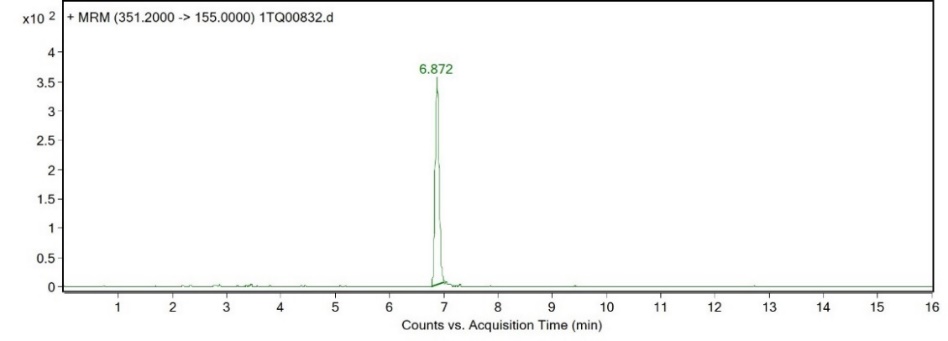 |
| **Figure 3.** Extracted chromatograms for (a) the transition m/z 384.3 → 197.1, and (b) the transition m/z 351.2 → 155.0. A sample of serum spiked with 10 ng/mL JWH-182 and IS was extracted and analyzed by LC-ESI-MS/MS. | |

Representative extracted chromatograms for a calibrator sample in which blank rat serum was spiked with 10 ng/mL of JWH-182 and internal standard (IS, JWH-018-d9) are presented in Figure 3.

**Table 3.** Matrix effect (ME), recovery (RE), process efficiency (PE) data for JWH-182 in one lot of blank rat serum.

| **Validation parameter** | **Nominal concentration (ng/mL serum)** | | | |
| --- | --- | --- | --- | --- |
|  | **1** | **10** | **100** | **1000** |
| ME (%) | 222 | 214 | 125 | 137 |
| RE (%) | 78 | 95 | 109 | 112 |
| PE (%) | 173 | 203 | 136 | 154 |

**2. Quantitation of JWH-182 in mice serum - Results**


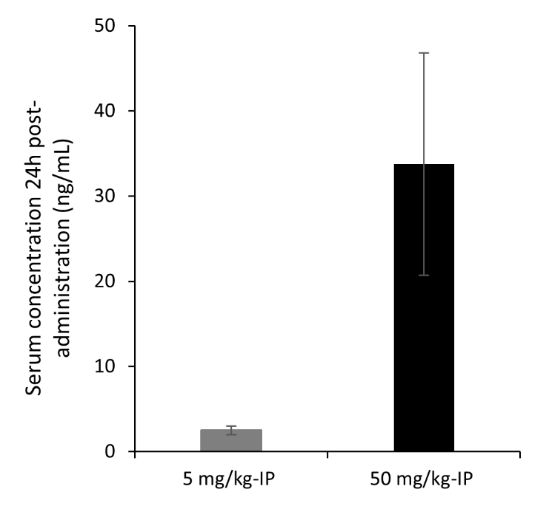


**Figure 4.** JWH-182 serum concentration at 24 hours after two doses administration. Results represent the mean ± standard error of the mean of three samples collected from 3 mice.

**3. Histopathological aspects of the organs collected in the acute toxicity study.**


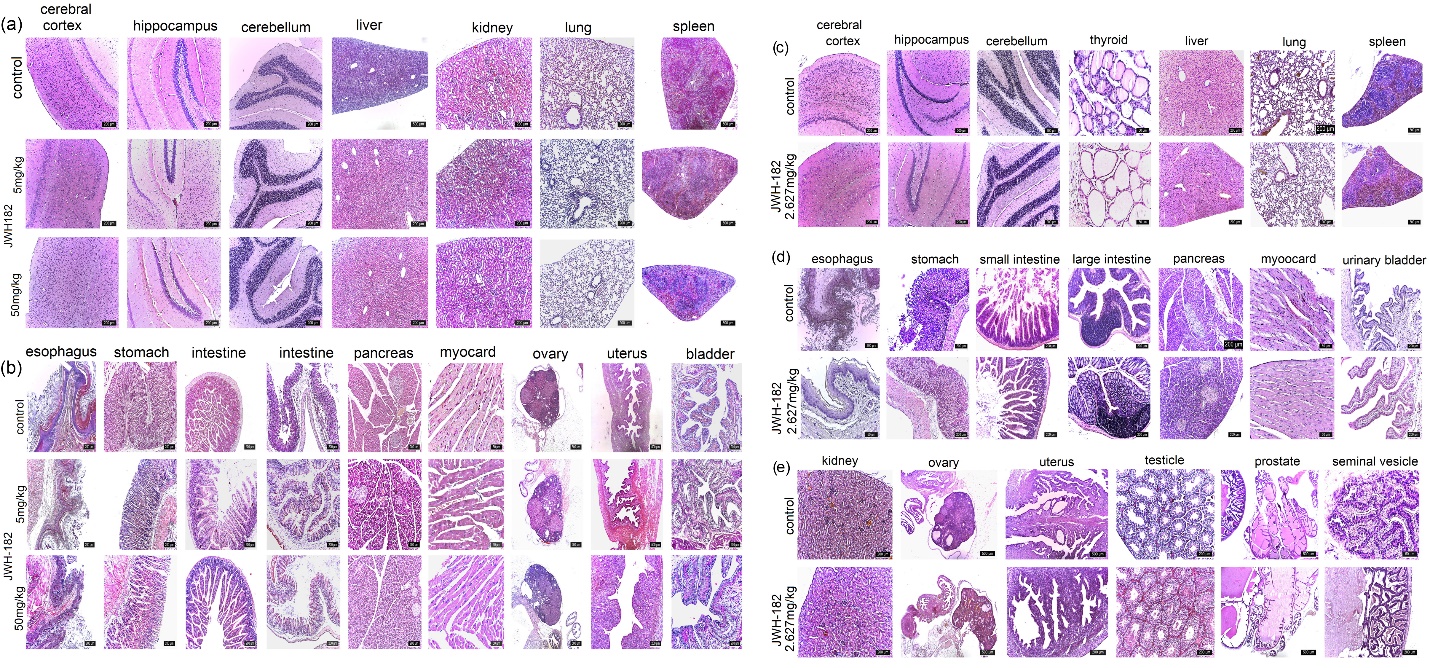


**Figure 5. Histopathological aspects of the organs collected in the acute toxicity study.**

(a) Histological aspects of the cerebral cortex, hippocampus, cerebellum, liver, kidney, lung, and spleen in mice exposed to JWH-182 acutely at doses of 0 mg/kg, 5 mg/kg, and 50 mg/kg. There were no histological changes observed using the standard hematoxylin and eosin (H&E) staining protocol. (b) Morpho-structure of the esophagus, stomach, small and large intestine, pancreas, myocardia, ovary, uterus and bladder in mice acutely exposed to 0 mg/kg 5 mg/kg, 50 mg/kg of JWH-182. Regardless of the dose, the organs studied showed no histological changes. Coloration in H&E; (c) Histological aspects of the cerebral cortex, hippocampus, cerebellum, thyroid, liver, lung and spleen in mice chronically exposed to JWH-182 at 2.627 mg/kg and control group. The organs studied showed no histological changes; H&E coloration; (c) Histological aspects of the esophagus, small and large intestine, pancreas, myocardia and urinary bladder in mice chronically exposed to 2.672 mg/kg and 0 mg/kg groups. As can be observed in the image, no histological changes were observed, hematoxylin and eosin (H&E) standard staining protocol; (e) Histological aspects of the kidney, ovary, uterus, testicle, prostate and seminal vesicle in mice chronically exposed to 2.672 mg/kg and 0 mg/kg groups. No histological changes occurred regardless of the dose, H&E coloration.

**References**

1. Hudson, S.; Ramsey, J.; King, L.; Timbers, S.; Maynard, S.; Dargan, P.I.; Wood, D.M. Use of High-Resolution Accurate Mass Spectrometry to Detect Reported and Previously Unreported Cannabinomimetics in “Herbal High” Products. *J. Anal. Toxicol.* **2010**, *34*, 252–260, doi:10.1093/jat/34.5.252.

2. Ambroziak, K.; Adamowicz, P. Simple screening procedure for 72 synthetic cannabinoids in whole blood by liquid chromatography–tandem mass spectrometry. *Forensic Toxicol.* **2018**, *36*, 280–290, doi:10.1007/s11419-017-0401-x.

3. Giorgetti, A.; Barone, R.; Pelletti, G.; Garagnani, M.; Pascali, J.; Haschimi, B.; Auwärter, V. Development and validation of a rapid LC-MS/MS method for the detection of 182 novel psychoactive substances in whole blood. *Drug Test. Anal.* **2022**, *14*, 202–223, doi:10.1002/dta.3170.

4. Matuszewski, B.K.; Constanzer, M.L.; Chavez-Eng, C.M. Strategies for the assessment of matrix effect in quantitative bioanalytical methods based on HPLC-MS/MS. *Anal. Chem.* **2003**, *75*, 3019–3030, doi:10.1021/ac020361s.

5. Filipiuc, L.-E.; Ştefănescu, R.; Solcan, C.; Ciorpac, M.; Szilagyi, A.; Cojocaru, D.; Stanciu, G.D.; Creangă, I.; Caratașu, C.-C.; Ababei, D.-C.; et al. Acute Toxicity and Pharmacokinetic Profile of an EU-GMP-Certified Cannabis sativa L. in Rodents. *Pharmaceuticals* **2023**, *16*, 694, doi:10.3390/ph16050694.
